# Mental health and risk behaviors of children in rural China with different patterns of parental migration: a cross-sectional study

**DOI:** 10.1186/s13034-019-0298-8

**Published:** 2019-10-22

**Authors:** Feng Wang, Jingjing Lu, Leesa Lin, Xudong Zhou

**Affiliations:** 1 The Institute of Social and Family Medicine, School of Medicine, Zhejiang University, Hangzhou, Zhejiang People’s Republic of China; 2 Faculty of Public Health Policy, London School of Hygiene & Tropical Medicine, Kings Cross, London, UK; 3 Department of Global Health and Social Medicine, Harvard Medical School, Boston, USA

**Keywords:** Left-behind children, Mental health, Risk behaviors, China, Rural–urban migration

## Abstract

**Background:**

One in seven members of China’s population are migrants. There are an estimated 41 million children left behind in rural areas who are living without one or both of their parents. The impact of two- and single-parent migration on child mental health and risk behaviors is unclear. The aim of this study was to compare the mental health and risk behaviors among children whose parents are either both migrating (B-LBC), have one parent migrating (O-LBC) or those whose parents do not migrate (N-LBC).

**Methods:**

This study was a cross-sectional survey using a self-administered questionnaire conducted in rural areas with high proportions of left behind children (LBC) in Anhui Province, southeast China. The tools used were the Strength and Difficulties Questionnaires, Youth Risk Behavior Survey and the Young’s Internet Addiction Test for Chinese.

**Results:**

Full data were available for 699 B-LBC, 552 O-LBC and 741 N-LBC. After adjusting for gender, age, grade, number of siblings and self-rated socio-economic status, B-LBC were significantly more likely to have higher emotional symptoms scores (B(SE) = 0.36(0.11), p < 0.01), higher hyperactivity scores (B(SE) = 0.22(0.11), p < 0.01) and higher total difficulties scores (B(SE) = 0.79(0.29), p < 0.01) than N-LBC. B-LBC were also more likely to be an addicted internet user (OR(95%CI) = 1.91(1.33, 2.76), p < 0.01) compared to N-LBC. However, there were no identified differences between O-LBC and N-LBC or between O-LBC and B-LBC in any measures.

**Conclusions:**

Our findings found that living with one parent or both parents was associated with better mental health and fewer risk behaviors than was being separated from both parents. Future research is needed to consider the implications of these findings for policies and programs to protect LBC, especially for those with two migrating parents.

## Background

Over the past decades, many workers originating from developing countries have relocated in search of better employment opportunities and other sources of income, migrating either internationally or internally within their home country (e.g., rural–urban migration). The majority of these migrants are employed in low-skilled jobs and living in poor conditions. Many migrants leave their children behind in the care of other family members or relatives while travelling, and thus the number of these so-called left-behind children (LBC) is high in many low- and middle-income countries [1]. Migrants are unable to bring their children with them for many reasons, including stringent entry policies, financial constraints, and limited access to public goods in the migrants’ destination cities [2].

China represents an emblematic case where massive rural–urban migration has resulted in an estimated 41 million children aged 18 years or younger who were left behind in rural areas, accounting for 29% of all rural children and 15% of the total child population in China [3]. Nearly half of LBC, roughly 20 million children have both parents migrating, with over 13 million and 8 million having only their father or mother migrating, respectively [3]. In China, the number of migrants has steadily increased over the past three decades, from 50 million in 1990 to 244 million in 2017, this accounts for roughly 31% of the entire working population [4].

The impact of parental migration on the mental health of LBC has drawn great attention from researchers across many disciplines (e.g., psychology, sociology, education, anthropology). It has been hypothesized that migration affects the well-being of children through the trade-off between an increase in family income and a decrease in parental care. For example, parents who migrate for work may increase family income and offer better education opportunities for their children, but parental absences may decrease care and stimulation, leading to a range of psychological and developmental risks [5, 6]. Previous studies have found that parental migration is a factor strongly related to depression and anxiety [7–12], loneliness [13, 14], low quality of life [15], low self-esteem [16], suicidal ideation and a range of behavioral problems [17–20].

Most existing studies treat LBC as a single group. Less attention, however, has been paid to the differences between children with both parents migrating and children with only one migrating parent. Although a small number of studies have evaluated such differences, the results from such studies remain mixed. While some studies have found that the prevalence of depressive symptoms was significantly higher among LBC with two migrating parents, compared to LBC with a single migrating parent [7, 9, 21]. Others have found the prevalence of anxiety disorders to be higher among children without migrating parents than it was for children living with one or neither parent [21, 22]. Giving these mixed findings on the consequences of different patterns of parental migration on children’s mental health outcomes, further studies are needed.

Less is known about the impact of parental migration on children’s risk behaviors. Previous research has indicated that parental and familial factors contribute to healthy development among children, and that a stable family environment is the primary source for the transmission of basic social, cultural and biological factors that may affect individual differences in risk behaviors [23–25]. However, current studies have failed to generate consistent findings with regard to the impact of parental migration on the risk behaviors of LBC. Negative impacts, such as internet addiction and binge drinking, have been documented [18, 19, 26, 27]. One study however, found no difference in problem behaviors between LBC and non-LBC in two Chinese provinces [28]. Risk behaviors manifesting during adolescence, such as smoking tobacco, drinking alcohol and internet addiction, may perpetuate into adulthood and have lasting adverse health effects [25, 29]. Given the large number of young Chinese netizens and a growing rate of internet overuse, it is urgent to examine addictive internet use among young children. Internet addiction, also known as pathological or addictive internet use, refers to “an incontrollable online compulsion under no influence of addictive substances” [30]. It was officially included into the fifth edition of the Diagnostic and Statistical Manual of Mental Disorders [31]. According to the China Internet Network Information Center (CNNIC), there were over 829 million netizens as of December, 2018 [32]. Of these netizens, more than one-fifth or approximately 169 million were young children less than 18 years of age. These young children spent 27.6 h per week online [32]. There is currently a dearth of information concerning the risk behaviors of children as differentiated by patterns of parental migration in China.

The major objective of this research was to investigate the effects of diverse forms of parental migration (including children with both parents migrating, those with one parent migrating and those with no migrating parents) on the mental health (including emotional symptoms, conduct problems, hyperactivity, peer problems and pro-social behaviors) and risk behaviors (including smoking, drinking and internet addiction) of children in rural China.

## Methods

### Sample

This study was a cross-sectional survey using self-completed questionnaires. Data in this study was collected from two counties in Anhui, a relatively underdeveloped south-east province in China. In 2018 Anhui ranked 22nd in GDP per capital among all 31 provinces, municipalities, and autonomous regions in Mainland China [33]. The two counties (Nanling and Wuwei) were selected in the rural region of Anhui.

For ease of sampling, we aimed to select areas where there were large numbers of LBC. To ascertain this we interviewed officials at the relevant departments of country or township governments to identify towns with high proportions of LBC. Four selected townships in southeast Anhui were included in this study. In each selected township, two schools with the highest proportions of LBC students were included in this study.

We established the sample size based on our previous study in rural China [34], with a power of 80% with a two-sided significance level of 0.05, which resulted in a total sample size of 2061. To be eligible for this study, students needed to be in Year 5 to Year 8 (mostly including children aged 11 to 17) from the selected schools. Children were excluded if one or both of their parents were deceased or if they lived in single-parent families.

Ethical approval for this study was obtained from Zhejiang University and local approvals were obtained from individual head teachers. Before the survey, informed consent was obtained from both the eligible children and their parents or legal guardians (through a letter sent home). All eligible students were provided with a detailed description of the study design. Those who agreed to participate were invited to complete a self-administered questionnaire in their classroom without a teacher present. Participants were told that they could refuse to fill out any items and could stop at any point. They were also told that there were no right or wrong answers, and that their answers would remain confidential. No one except the researchers would have access to information written in the questionnaire.

### Measures

#### Demographic characteristic

Demographic characteristic that were collected included gender, age, grade, and number of siblings. As it would be difficult for children to report their parents’ annual individual or household income, we asked about perceived comparative wealth in the community: “How do you feel your household wealth compares with the average in your community (much better off/better off, the same, poorer/much poorer)?”

#### Parental migration status

Parental migration status was determined according to two questions: “has your father (and your mother) migrated into other places for work and been absent for over 6 months?” The options were “yes, currently migrate”, “yes, previously migrate”, and “no, never”. If both parents were currently migrating, the child was defined as a “B-LBC”; if not, and if one parent was currently migrating, the child was defined as a “O-LBC”; and if neither parents had migrated, the child defined as a “N-LBC”.

#### Mental health

Child mental health was assessed with the Chinese student version of the Strength and Difficulties Questionnaires (SDQ) [35–37]. The SDQ comprises 25 items and is scored on a 3-point Likert scale (0 = not true, 1 = somewhat true, 2 = certainly true). It has five dimensions, each with 5-items: emotional symptoms, conduct problems, hyperactivity, peer problems and pro-social behaviors. Each dimension was measured by the summed score of the five items as a subscale, with values ranging from 0 to 10. All but the pro-social subscale were then grouped together to generate a total difficulties score, ranging from 0 to 40. In all dimensions but pro-social, higher scores indicate more severe difficulties. Scores can be analyzed as individual subscale and by a total, as categorical or continuous variables. The categorical variables were grouped into three categories: “abnormal”, “borderline”, or “normal” categories. The cut-offs of “abnormal” of the total difficulties and the five subscales are as follows: total difficulties (≥ 20), emotional symptoms (≥ 7), conduct problems (≥ 5), hyperactivity (≥ 7), peer problems (≥ 6) and pro-social behaviors (≤ 4). The validity of the SDQ has been well-established in the Chinese context [35, 37]. The Cronbach α were from 0.644 to 0.938 for each subscale in this study.

#### Smoking and drinking

Specific questions on risk behaviors were measured by a scale of five items adapted from the Youth Risk Behavior Survey (YRBS) [38]. We focused on the aspects that better apply to rural children in China. The questions asked were: (1) Have you ever tried cigarette smoking, even one or two puffs? (2) During the past 30 days, on how many days did you smoke cigarettes? (3) Have you ever had at least one drink of alcohol other than a few sips? (4) During the past 30 days, on how many days did you have at least one drink of alcohol? (5) During the past 30 days, how many times had you ever been sick or had uncomfortable reactions after you had alcohol?

#### Internet addiction

Internet addiction was assessed using the (YIAT-C) Young’s Internet Addiction Test for Chinese [39, 40]. The scale is a 20-item tool where participants rank certain statements along a 5-point continuum from “not at all” to “always”. Internet addiction was measured by summing the scores of all items (thus ranging from 20 to 100). Scores can be analyzed as continuous or categorical variables, the latter divisible into “normal”, “low”, “mild”, or “severe” categories, corresponding to scores of 20–40, 41–60, 61–80 and 81 and over [39]. The YIAT-C has proven its reliability and validity across different cultures and settings, and has been validated in the Chinese context [39, 41]. The Cronbach α of this scale was 0.917 in the study.

### Statistical analysis

Chi-square test and analyses of variance were conducted to compare sample characteristics and dependent variables among three groups of children with different parental migration status. The Scheffe test (for continuous variables) or Bonferroni test (for categorical variables) was applied in post hoc analyses that compared mental or behaviors outcomes across three parental migration groups. For those mental and behavioral indicators, which were significant in the univariate analysis, we controlled for gender, age, grade, number of siblings and self-rated socio-economic status using logistic or multiple linear regression models. Data management and analysis were performed using SPSS 24.0 for Windows.

## Results

The final study sample included 1922 participants, including 699 B-LBC, 552 O-LBC and 741 N-LBC. There were 27 outright refusals (1.3%) overall, and another 39 (1.9%) had to be discarded because of non-completion of key variables (parental migration status). Table 1 presents the socio-demographic characteristics of children by their parental migration status. Overall, there were more boys than girls in the study sample and the gender distribution did not differ across the three groups. The number of students from grade 7 to grade 8 was slightly higher in the O-LBC group than in the other two groups. In regards to household wealth, nearly one-fifth of O-LBC reported that they were from wealthier households, whereas the respective proportions for B-LBC and N-LBC were 27.8% and 28%. Approximately one-third of respondents were single children across the three groups.

**Table 1:** Demographic characteristics of the sample, n(%)

	B-LBC	O-LBC	N-LBC	F or χ^2^	p value
Gender				0.24	0.888
Male	380 (55.0)	298 (54.7)	394 (53.8)		
Female	311 (45.0)	247 (45.3)	339 (46.2)		
Age, mean (SD)	13.1 (1.2)	13.2 (1.2)	13.1 (1.2)	2.57	0.076
Grade				6.52	0.038
Grade5 Grade6	323 (46.2)	215 (39.0)	316 (42.7)		
Grade7 Grade8	376 (53.8)	336 (61.0)	424 (57.3)		
Perceived income level				12.69	0.013
Much better off/better off	193 (27.8)	124 (22.6)	205 (28.0)		
The same	455 (65.7)	363 (66.2)	471 (64.3)		
Poorer/much poorer	45 (6.5)	61 (11.1)	57 (7.8)		
Any sibling				2.54	0.281
Yes	249 (35.6)	182 (33.0)	235 (31.7)		
No	450 (64.4)	370 (67.0)	506 (68.3)		

*B-LBC* left-behind children with both parents migrating, *O-LBC* left-behind children with one parent migrating, *N-LBC* neither parents had migrated

Table 2 shows the differences between the three groups of children in terms of the key mental health outcomes from the SDQ, including total difficulties and the five subscales. B-LBC had higher emotional symptoms, hyperactivity and total difficulties mean scores than did N-LBC. No significant differences were identified between the O-LBC and N-LBC or between the O-LBC and B-LBC in total or all subscale scores according to post hoc tests. When analyzed as categorical variables, the proportion of abnormal emotional symptoms, hyperactivity and total difficulties score were found to be significantly more common in the B-LBC group, as shown in Table 2. It is important to note that B-LBC reported scores indicating abnormality in these three outcomes (12.5%, 14.0% and 13.2% respectively) at a rate of nearly 1.5 times what was observed in N-LBC (7.5%, 9.5% and 8.4% respectively). The frequencies of individual risk behaviors and internet addiction by child group are illustrated in Table 3. There were few differences in risk behaviors between the three groups. In general, B-LBC were more likely to have been sick or have uncomfortable reactions due to drinking than were the N-LBC group. Overall, B-LBC had a higher prevalence rates of addictive internet use than did N-LBC.

**Table 2:** Group differences in terms of Strength and Difficulties Questionnaires, mean/n [SD(%)]

	B-LBC (1)	O-LBC (2)	N-LBC (3)	F or χ^2^	PC^$^
Emotional symptoms^a^, mean (SD)	3.6 (2.2)	3.5 (2.2)	3.3 (2.1)	4.75**	(1, 3)
Emotional symptoms (categorical)^1^				10.58**	(1, 3)
Normal/borderline (0–6)	608 (87.5)	490 (88.9)	683 (92.5)		
Abnormal (7–10)	87 (12.5)	61 (11.1)	55 (7.5)		
Conduct problems^b^, mean (SD)	2.5 (1.6)	2.4 (1.6)	2.4 (1.6)	0.82	
Conduct problems (categorical)^2^				0.65	
Normal/borderline (0–4)	619 (89.1)	493 (90.5)	660 (89.6)		
Abnormal (5–10)	76 (10.9)	52 (9.5)	77 (10.4)		
Hyperactivity^c^, mean (SD)	4.1 (2.2)	4.0 (2.1)	3.8 (2.1)	3.64*	(1, 3)
Hyperactivity (categorical)^3^				8.31*	(1, 3)
Normal/borderline (0–6)	601 (86.0)	474 (86.3)	668 (90.5)		
Abnormal (7–10)	98 (14.0)	75 (13.7)	70 (9.5)		
Peer problems^d^, mean (SD)	2.7 (1.7)	2.7 (1.6)	2.6 (1.6)	0.98	
Peer problems (categorical)^4^				2.00	
Normal/borderline (0–5)	653 (93.4)	521 (94.6)	702 (95.1)		
Abnormal (6–10)	46 (6.6)	30 (5.4)	36 (4.9)		
Total difficulties score^e^, mean (SD)	12.8 (5.5)	12.7 (5.3)	12.0 (5.2)	4.25*	(1, 3)
Total difficulties score (categorical)^5^				8.53*	(1, 3)
Normal/borderline (0–19)	600 (86.8)	479 (88.7)	667 (91.6)		
Abnormal (20–40)	91 (13.2)	61 (11.3)	61 (8.4)		
Prosocial^f^, mean (SD)	6.9 (2.0)	6.8 (2.1)	7.0 (2.0)	2.03	
Prosocial (categorical)^6^				1.82	
Normal/borderline (5–10)	625 (89.9)	491 (89.1)	672 (91.3)		
Abnormal (0–4)	70 (10.1)	60 (10.9)	64 (8.7)		

*B-LBC* left-behind children with both parents migrating, *O-LBC* left-behind children with one parent migrating, *N-LBC* neither parents had migrated

* p < 0.05, **p  < 0.01

^$^PC indicate the significance of pairwise comparisons in the post hoc analysis

^a^Partial η^2^ = 0.005; ^b^Partial η^2^ = 0.001; ^c^Partial η^2^ = 0.004; ^d^Partial η^2^ = 0.001; ^e^Partial η^2^ = 0.004; ^f^Partial η^2^ = 0.002

1: Phi = 0.073; 2: Phi = 0.018; 3: Phi = 0.065; 4: Phi = 0.032; 5: Phi = 0.066; 6: Phi = 0.030

**Table 3:** Behaviors problems by parental migration groups, n (%)

	B-LBC (1)	O-LBC (2)	N-LBC (3)	χ^2^	PC^$^
Ever smoking				0.09	
Yes	130 (18.6)	104 (18.8)	135 (18.2)		
No	569 (81.4)	448 (81.2)	606 (81.8)		
Smoking at least 1 day during the 30 days before the survey				0.96	
0 days	108 (87.1)	85 (82.5)	107 (85.6)		
≥ 1 days	16 (12.9)	18 (17.5)	18 (14.4)		
Ever drinking				0.77	
Yes	278 (39.8)	217 (39.3)	278 (37.6)		
No	421 (60.2)	335 (60.7)	461 (62.4)		
Drinking at least 1 day during the 30 days before the survey				0.27	
0 days	188 (67.4)	149 (69.3)	190 (69.1)		
≥ 1 days	91 (32.6)	66 (30.7)	85 (30.9)		
Having been sick or had uncomfortable reactions after had drunk				9.19*	(1, 3)
0 times	249 (89.2)	200 (92.2)	265 (96.0)		
≥ 1 times	30 (10.8)	17 (7.8)	11 (4.0)		
Internet addiction				14.17*	(1, 3)
None (20–40)	251 (47.4)	188 (44.5)	279 (49.4)		
Low (41–60)	189 (35.7)	175 (41.5)	230 (40.7)		
Mild (61–80)	79 (14.9)	53 (12.6)	49 (8.7)		
Severe (81–100)	11 (2.1)	6 (1.4)	7 (1.2)		

*B-LBC* left-behind children with both parents migrating, *O-LBC* left-behind children with one parent migrating, *N-LBC* neither parents had migrated

*p < 0.05

^$^PC indicate the significance of pairwise comparisons in the post hoc analysis

Table 4 presents the regression results of SDQ subscales and total difficulties scores that showed significant between-group differences in Table 2. After adjusting for all covariates, B-LBC were significantly more likely to have higher emotional symptoms scores, higher hyperactivity scores and higher total difficulties scores than N-LBC. After adjusting for gender, age, grade, number of siblings and self-rated socio-economic status (Table 5), B-LBC were also more likely to have been sick or have uncomfortable reactions after had drunk and to be an addicted internet user.

**Table 4:** Linear regression analysis for SDQ (emotional symptoms, hyperactivity, total difficulties) by parental migration groups and demographic characteristic

	Emotional symptoms^a^ B(SE)	Hyperactivity^b^ B(SE)	Total difficulties^c^ B(SE)
Parental migration status (ref: N-LBC)			
B-LBC	0.36 (0.11)**	0.22 (0.11)**	0.79 (0.29)**
O-LBC	0.21 (0.12)	0.27 (0.12)	0.66 (0.31)
Gender (ref: male)			
Female	0.55 (0.10)***	0.08 (0.10)	0.17 (0.25)
Age	0.02 (0.04)	0.25 (0.04)***	0.16 (0.10)
Perceived income level (ref: much better off/better off/the same)			
Poorer/much poorer	0.47 (0.18)**	0.31 (0.18)	1.66 (0.44)***
Any sibling (ref: yes)			
No	− 0.18 (0.10)	− 0.26 (0.10)*	− 0.57 (0.26)*

*B-LBC* left-behind children with both parents migrating, *O-LBC* left-behind children with one parent migrating, *N-LBC* neither parents had migrated

*p < 0.05, **p < 0.01, ***p < 0.001

^a^N = 1932, R^2^ = 0.027; ^b^N = 1934, R^2^ = 0.028; ^c^N = 1907, R^2^ = 0.016

**Table 5:** Logistic regression analysis for risk behaviors by parental migration groups and demographic characteristic, OR (95% CI)

	Smoking^a^	Drinking^b^	Feeling sick^c^	Internet addiction^d^
Parental migration status (ref: N-LBC)				
B-LBC	1.04 (0.79, 1.37)	1.14 (0.91, 1.42)	3.15 (1.51, 6.61)**	1.91 (1.33, 2.76)**
O-LBC	1.03 (0.77, 1.38)	1.06 (0.84, 1.34)	2.22 (0.99, 4.96)	1.33 (0.89, 1.98)
Gender (ref: male)				
Female	0.59 (0.47, 0.76)***	0.80 (0.66, 0.96)*	1.53 (0.85, 2.74)	0.75 (0.55, 1.03)
Age	1.34 (1.21, 1.48)***	1.34 (1.24, 1.45)***	0.98 (0.78, 1.22)	1.48 (1.29, 1.69)***
Perceived income level (ref: much better off/better off/the same)				
Poorer/much poorer	1.20 (0.77, 1.74)	0.84(0.60, 1.19)	0.96 (0.33, 2.82)	1.20 (0.68, 2.11)
Any sibling (ref: yes)				
No	0.83 (0.65, 1.07)	0.89 (0.73, 1.08)	0.92 (0.51, 1.66)	0.80 (0.57, 1.12)

*B-LBC* left-behind children with both parents migrating, *O-LBC* left-behind children with one parent migrating, *N-LBC* neither parents had migrated

*p < 0.05, **p < 0.01, ***p < 0.001

^a^Have you ever tried cigarette smoking, even one or two puffs? (0 = No; 1 = Yes)

^b^Have you ever had a drink of alcohol, other than a few sips? (0 = No; 1 = Yes)

^c^During the past 30 days, how many times have you ever been sick or had uncomfortable reactions after had alcohol? (0 = 0 times; 1 = ≥ 1 times)

^d^Internet addiction (0 = none and low; 1 = mild and severe)

## Discussion

The present study was designed to determine the effects of different patterns of parental migration on the mental health and risk behaviors of children in rural China. We found that after controlling for the major confounders of gender, age, grade, number of siblings and self-rated socio-economic status, B-LBC were significantly more likely to have higher levels of emotional symptoms, hyperactivity and higher total difficulties than N-LBC. Furthermore, a higher proportion of B-LBC reported having been sick or having uncomfortable reactions after had drunk and addictive internet use when compared to their N-LBC counterparts, with strong and consistent associations.

A number of limitations on this study need to be considered. Firstly, while the sample size is large, it is taken from just one province in south-east China, so it is inappropriate to extrapolate the results of this study to the whole country. Nonetheless, this province does resemble a number of Chinese provinces with large populations of LBC, such as Henan, Sichuan, Guizhou and Guangdong. Secondly, the findings are limited by the use of a cross sectional design. More research is needed to explore these issues using longitudinal analysis. Thirdly, due to the small sample size of mother-only migration (4.2%), we could not assess differences in mental health and risk behaviors between father-only migration and mother-only migration. In the future, it would be helpful for research to distinguish between father- and mother-migration in these outcomes. Fourthly, the current research has only examined a limited number of potential determinants. Other possible variables that were not included in this research were children’s caretaking arrangements, family social capital, etc. Lastly, we used child self-reported data only. We were unable to collect data from other sources (e.g., parents, caregivers and teachers) due to practical constraints in recruiting migrant parents and lack of literacy in some grandparents.

Despite these limitations, the findings from this study make several contributions to the current literature. Firstly, we confirm previous findings that children with two migrating parents reported the worst mental health outcomes among the three groups of rural children [21, 42]. However, children with one parent migrating had a similar prevalence rate of mental disorders to children living with both parents [43]. There are several possible explanations for this result. Migrating parents may provide more economic resources via remittances for their children that may be beneficial for the children’s development in two migrating parents families. However, both paternal and maternal absences can be harmful due to factors such as reduced parental supervision and weakened parent–child bonding and communication [18, 44]. Children with only one parent migrating may enjoy better financial conditions as a result of their parents’ earnings and also benefit from staying with one of their parents. Overall, as illustrated by the findings, children with two migrating parents could be more vulnerable than those with only one migrating parent or no migrating parents.

Secondly, the SDQ has been widely used as a screening tool for psychiatric disorders in children, and those who scored as “abnormal” may need further psychological assessment. The results of this study indicate that 13.2% of B-LBC and 11.3% of O-LBC fall into the “abnormal” total difficulties category, this should be of great concern, given that such psychological difficulties are not well identified. This is also clearly an issue worth considering due to the massive size of the LBC population in China.

Thirdly, this study did not find any significant differences in the proportions of children who have ever smoked or consumed alcohol, which is consistent with existing studies [34]. A possible explanation for these results may relate to the more traditional views of childhood in China. Prior studies conducted in China have demonstrated that these risk behaviors tend to start after children leave school in both urban and rural settings [45]. Some authors speculate that even when one or both parents are migrating for work, children are left in the care of their parents who stay at home, grandparents or other relatives. It seems possible that both the primary caregiver and the parents working away from home prioritize the prevention of children developing externalizing behavioral problems over the promotion of children’s psychological well-being [28]. However, we observed a higher prevalence of feeling sick or having uncomfortable reactions after drinking and internet addiction amongst B-LBC compared to their N-LBC counterparts. These may be partly due to a lack of parental supervision and care [26]. However, more research on this topic should be done before the association between parental migration status and children’s risk behaviors (especially “internet addiction”) is more clearly understood. Care strategies and interventions need to be developed for children with high externalizing problems.

Our findings also presented demographic influences. It is noteworthy that associations of age, gender and household wealth level with mental health and behavior outcomes differed across multiple dimensions. Importantly, our results indicated that girls were much more vulnerable to emotional distress. In comparison to boys, girls were at a significantly lower risk for ever smoking, which suggested that boys might express problems more externally whereas girls might express internally. This is similar with previous studies conducted in rural China [46, 47]. Girls in rural China were more likely than boys to be responsible for younger siblings and household chores after their parents left. Feeling less “preferred” in the household and then being left behind may be particularly damaging to the rural girls’ emotional well-being, especially during the years around puberty.

Our findings strongly suggests that LBC, especially those with two migrating parents, have markedly higher psychological and behavioral difficulties, independent of individual and family circumstances. Given our results, the observance of a relative decrease in LBC is encouraging. The number of LBC has decreased dramatically over the last 10 years, decreasing from 58 million to 41 million between 2005 and 2015 [3, 48]. This aligns with the Chinese government’s policies to provide better care and protection to LBC. The State Council of China issued a set of guidelines which lay out measures to gradually decrease the number of LBC [49]. The government provided greater assistance, such as granting families of migrant workers urban citizenship or subsidies in housing or education. Rural migrant workers are also encouraged to return to their hometown and start their own businesses. However, at the current level of 41 million children, the negative impacts of parental migration on children is still a huge challenge in China.

The key question is what can be done to support the millions of LBC who have high psychological difficulties. According to the latest report, there are currently 1.85 psychiatrists and 3.77 psychiatric nurses per 100,000 people in China [50]. However, the overwhelming majority of mental health services are located at county level and above. At present, doctors from township and village are not trained to identify and treat mental health problems. On a positive note, China is currently undergoing a major reform process aimed at developing mental health service systems for population across sociodemographic groups by year 2025. Addressing the shortcomings of rural mental health services and training school teachers to identify key symptoms or signs of mental illness have been included in this process. Now, with more resources available for mental health services, there is a real opportunity to support the most vulnerable LBC though the success has yet to be evaluated. However, an increasing number of models of community-based interventions are emerging, including our own intervention [51]. Our intervention program, which takes a community care approach, featured “children’s clubs” run by local residents that provided activities, support, and a safe social place for LBC in their home villages [51]. Our intervention outcomes indicated the success in establishing a community care platform to benefit the emotional and behavioral well-being of LBC, and to enhance the community support networks.

## Conclusions

In conclusion, this work explored the differences between children with two migrating parents and children with one migrating parent or with neither parent migrating, thus extending existing knowledge on LBC who have been previously treated as a single group. The evidence from this study suggests that LBC with both parents migrating are the most vulnerable children who engage in higher rates of risk behaviors and are more likely to have psychological difficulties. Our results also imply that the mental health and risk behaviors were similar in the O-LBC and N-LBC group. Taken together, these results suggest that further support and care from local mental health services and community need to be provided for children with two migrating parents.

## Data Availability

The datasets used during the current study are available from the corresponding author on reasonable request.

## Abbreviations

LBC: left-behind children
B-LBC: left-behind children with both parents migrating
O-LBC: left-behind children with one parent migrating
N-LBC: neither parents had migrated

## References

[1] Nobles J (2013). Migration and father absence: shifting family structure in Mexico. Demography.

[2] Peng X (2011). China’s demographic history and future challenges. Science.

[3] Lv L, Yan F, Duan C, Cheng M (2018). Changing patterns and development challenges of child population in China. Popul Res.

[4] National Health Commission of the People’s Republic of China. China migration population development report 2018. 2018. http://www.nhc.gov.cn/wjw/xwdt/201812/a32a43b225a740c4bff8f2168b0e9688.shtml. Accessed 2 Mar 2019.

[5] Wang L, Mesman J (2015). Child development in the face of rural-to-urban migration in China: a meta-analytic review. Perspect Psychol Sci..

[6] Lu Y (2012). Household migration, social support, and psychosocial health: the perspective from migrant-sending areas. Soc Sci Med.

[7] Wang L, Feng Z, Yang G, Yang Y, Dai Q, Hu C (2015). The epidemiological characteristics of depressive symptoms in the left-behind children and adolescents of Chongqing in China. J Affect Disord.

[8] Zhao X, Chen J, Chen MC, Lv XL, Jiang YH, Sun YH (2014). Left-behind children in rural China experience higher levels of anxiety and poorer living conditions. Acta Paediatr.

[9] He B, Fan J, Liu N, Li H, Wang Y, Williams J (2012). Depression risk of ‘left-behind children’ in rural China. Psychiatr Res.

[10] Cheng J, Sun YH (2015). Depression and anxiety among left-behind children in China: a systematic review. Child Care Health Dev.

[11] Zhao J, Li Q, Wang L, Lin L, Zhang W (2019). Latent profile analysis of left-behind adolescents’ psychosocial adaptation in rural china. J Youth Adolesc.

[12] Chang F, Jiang Y, Loyalka P, Chu J, Shi Y, Osborn A (2019). Parental migration, educational achievement, and mental health of junior high school students in rural China. China Econ Rev.

[13] Liu LJ, Sun X, Zhang CL, Wang Y, Guo Q (2010). A survey in rural China of parent-absence through migrant working: the impact on their children’s self-concept and loneliness. BMC Public Health.

[14] Jia Z, Tian W (2010). Loneliness of left-behind children: a cross-sectional survey in a sample of rural China. Child Care Health Dev.

[15] Huang Y, Zhong X, Li Q, Xu D, Zhang X, Feng C (2015). Health-related quality of life of the rural-China left-behind children or adolescents and influential factors: a cross-sectional study. Health Qual Outcomes..

[16] Luo J, Wang LG, Gao WB (2012). The influence of the absence of fathers and the timing of separation on anxiety and self-esteem of adolescents: a cross-sectional survey. Child Care Health Dev.

[17] Fellmeth G, Rose-Clarke K, Zhao C, Busert LK, Zheng Y, Massazza A (2018). Health impacts of parental migration on left-behind children and adolescents: a systematic review and meta-analysis. Lancet.

[18] Wen M, Lin D (2012). Child development in rural China: children left behind by their migrant parents and children of non-migrant families. Child Dev.

[19] Gao Y, Li L, Chan EYY, Lau J, Griffiths SM (2013). Parental migration, self-efficacy and cigarette smoking among rural adolescents in south China. PLoS ONE.

[20] Palos-Lucio G, Flores M, Rivera-Pasquel M, Salgado-de-Snyder VN, Monterrubio E, Henao S (2015). Association between migration and physical activity of school-age children left behind in rural Mexico. Int J Public Health..

[21] Shen M, Gao J, Liang Z, Wang Y, Du Y, Stallones L (2015). Parental migration patterns and risk of depression and anxiety disorder among rural children aged 10-18 years in China: a cross-sectional study. BMJ Open.

[22] Tao XW, Guan HY, Zhao YR, Fan ZY (2014). Mental health among left-behind preschool-aged children: preliminary survey of its status and associated risk factors in rural China. J Int Med Res.

[23] Piko BF, Fitzpatrick KM (2007). Socioeconomic status, psychosocial health and health behaviours among Hungarian adolescents. Eur J Public Health..

[24] Wang J, Hughes J, Murphy GT, Rigby JA, Langille DB (2003). Suicidal behaviours among adolescents in northern Nova Scotia. Can J Public Health.

[25] Riesch SK, Anderson LS, Krueger HA (2006). Parent–child communication processes: preventing children’s health-risk behavior. J Spec Pediatr Nurs..

[26] Gao Y, Li LP, Kim JH, Congdon N, Lau J, Griffiths S (2010). The impact of parental migration on health status and health behaviours among left behind adolescent school children in China. BMC Public Health..

[27] Feng H, Liu J, Wang Y, He G (2011). Sociodemographic correlates of behavioral problems among rural Chinese schoolchildren. Public Health Nurs.

[28] Liu Y, Li X, Chen L, Qu Z (2015). Perceived positive teacher-student relationship as a protective factor for Chinese left-behind children’s emotional and behavioural adjustment. Int J Psychol..

[29] Spear HJ, Kulbok PA (2001). Adolescent health behaviors and related factors: a review. Public Health Nurs.

[30] Young K, Pistner M, O’Mara J, Buchanan J (1999). Cyber disorders: the mental health concern for the new millennium. Cyberpsychol Behav..

[31] American Psychological Association (2013). Diagnostic and statistical manual of mental Disorders-V (DSM-5).

[32] China Internet Network Information Center. The statistic report of the development of China internet network, 2018. 2019. http://www.cnnic.net.cn/hlwfzyj/hlwxzbg/hlwtjbg/201902/P020190318523029756345. Accessed 7 Aug 2019.

[33] National Bureau Of Statistics Of China. Statistical Communiqué of the People’s Republic of China on the 2018 National Economic and Social Development. 2019. http://www.stats.gov.cn/tjsj/zxfb/201902/t20190228_1651265.html. Accessed 2 Mar 2019.

[34] Wang F, Zhou X, Hesketh T (2017). Psychological adjustment and behaviours in children of migrant workers in China. Child Care Health Dev.

[35] Goodman R (1997). The strengths and difficulties questionnaire: a research note. J Child Psychol Psychiatry.

[36] Goodman R (1999). The extended version of the strengths and difficulties questionnaire as a guide to child psychiatric caseness and consequent burden. J Child Psychol Psychiatry.

[37] Kou J, Du Y, Xia L (2007). Formulation of children strength and difficulties questionnaire (the edition for students) for Shanghai norm. China J Health Psychol.

[38] Kann L, Warren CW, Harris WA, Collins JL, Douglas KA, Collins ME (1995). Youth risk behavior surveillance–United States, 1993. MMWR CDC Surveill Summ.

[39] Ni X, Yan H, Chen S, Liu Z (2009). Factors influencing internet addiction in a sample of freshmen university students in China. Cyberpsychol Behav..

[40] Young KS (1998). Internet addiction: the emergence of a new clinical disorder. Cyberpsychol Behav..

[41] Khazaal Y, Billieux J, Thorens G, Khan R, Louati Y, Scarlatti E (2008). French validation of the internet addiction test. Cyberpsychol Behav..

[42] Su S, Li X, Lin D, Xu X, Zhu M (2013). Psychological adjustment among left-behind children in rural China: the role of parental migration and parent-child communication. Child Care Health Dev.

[43] Zhao J, Liu X, Wang M (2015). Parent-child cohesion, friend companionship and left-behind children’s emotional adaptation in rural China. Child Abuse Negl.

[44] Givaudan M, Pick S (2013). Children left behind: how to mitigate the effects and facilitate emotional and psychosocial development. Child Abuse Negl.

[45] Hesketh T, Ding QJ, Tomkins A (2001). Smoking among youths in China. Am J Public Health.

[46] Fan F, Su L, Gill MK, Birmaher B (2010). Emotional and behavioral problems of Chinese left-behind children: a preliminary study. Soc Psychiatry Psychiatr Epidemiol.

[47] Hu H, Lu S, Huang C (2014). The psychological and behavioral outcomes of migrant and left-behind children in China. Child Youth Serv Rev..

[48] Duan C (2015). Several key issues related with migrant children and left-behind children. China Agric Univ J Soc Sci Ed.

[49] The State Council of China. Strengthening the care and protection for rural left-behind children. 2016. http://www.gov.cn/zhengce/content/2016-02/14/content_5041066.htm. Accessed 2 Mar 2019.

[50] National Health Commission of the People’s Republic of China. Medical and health services in China. 2014. http://en.nhc.gov.cn/2014-06/13/c_45997.htm. Accessed 2 Mar 2019.

[51] Zhao C, Zhou X, Wang F, Jiang M, Hesketh T (2017). Care for left-behind children in rural China: a realist evaluation of a community-based intervention. Child Youth Serv Rev..

